# Inhibiting YAP expression suppresses pancreatic cancer progression by disrupting tumor-stromal interactions

**DOI:** 10.1186/s13046-018-0740-4

**Published:** 2018-03-27

**Authors:** Zhengdong Jiang, Cancan Zhou, Liang Cheng, Bin Yan, Ke Chen, Xin Chen, Liang Zong, Jianjun Lei, Wanxing Duan, Qinhong Xu, Xuqi Li, Zheng Wang, Qingyong Ma, Jiguang Ma

**Affiliations:** 1 Department of Hepatobiliary Surgery, First Affiliated Hospital of Xi’an Jiaotong University, 277 West Yanta Road, Xi’an, 710061 China; 2 Department of General Surgery, First Affiliated Hospital of Xi’an Jiaotong University, 277 West Yanta Road, Xi’an, 710061 China; 3 Department of Anesthesiology, First Affiliated Hospital of Xi’an Jiaotong University, 277 West Yanta Road, Xi’an, 710061 China

**Keywords:** YAP, Pancreatic cancer, Pancreatic stellate cells, Invasion

## Abstract

**Background:**

Hippo/YAP pathway is known to be important for development, growth and organogenesis, and dysregulation of this pathway leads to tumor progression. We and others find that YAP is up-regulated in pancreatic ductal adenocarcinoma (PDAC) and associated with worse prognosis of patients. Activated pancreatic stellate cells (PSCs) forming the components of microenvironment that enhance pancreatic cancer cells (PCs) invasiveness and malignance. However, the role and mechanism of YAP in PDAC tumor-stromal interaction is largely unknown.

**Methods:**

The expression of YAP in Pancreatic cancer cell lines and PDAC samples was examined by Western blot and IHC. The biological role of YAP on cancer cell proliferation, epithelial-mesenchymal transition (EMT) and invasion were evaluated by MTT, Quantitative real-time PCR analysis, Western blot analysis and invasion assay. The effect of YAP on PSC activation was evaluated by PC-PSC co-culture conditions and xenograft PDAC mouse model.

**Results:**

Firstly, knockdown of YAP inhibits PDAC cell proliferation and invasion in vitro. In addition, YAP modulates the PC and PSC interaction via reducing the production of connective tissue growth factor (CTGF) from PCs, inhibits paracrine-mediated PSC activation under PC-PSC co-culture conditions and in turn disrupts TGF-β1-mediated tumor-stromal interactions. Lastly, inhibiting YAP expression prevents tumor growth and suppresses desmoplastic reaction in vivo.

**Conclusions:**

These results demonstrate that YAP contributes to the proliferation and invasion of PC and the activation of PSC via tumor-stromal interactions and that targeting YAP may be a promising therapeutic strategy for PDAC treatment.

**Electronic supplementary material:**

The online version of this article (10.1186/s13046-018-0740-4) contains supplementary material, which is available to authorized users.

## Background

Pancreatic ductal adenocarcinoma (PDAC) is the fourth most common cause of cancer-related death in the USA, with a 5-year survival rate of less than 7% and a median survival time of less than 6 months. There were an estimated 53,070 new diagnoses and 41,780 deaths from pancreatic cancer in the United States in 2015 [1]. Pancreatic cancer is a highly invasive and metastatic cancer, and these characteristics are primarily responsible for treatment failure and the poor clinical prognosis. Pancreatic tumors are surrounded by a dense desmoplastic stroma [2] that consists of pancreatic stellate cells (PSC), immune cells, lymphatic and vascular endothelial cells, pathologically increased nerves and extracellular matrix (ECM), which create a complex tumor microenvironment that promotes pancreatic cancer development, invasion, metastasis and resistance to chemotherapy [3].

PSCs are the major cellular contributors to the desmoplastic reaction in PDAC and are thought to play an important role in the pathobiology of pancreatitis and pancreatic cancer [4]. Under normal conditions, PSC are maintained in a “quiescent” state; however, they can switch to a more “immature” phenotype that is characterized by a tendency to synthesize certain biologically active molecules (such as matrix metalloproteinase (MMP)-2, MMP-9, and transforming growth factor (TGF)-β1) in response to various stimuli [5]. PSC are activated by direct contact with pancreatic cancer cells (PCs) or paracrine cytokines produced by PCs, including sonic Hedgehog (SHH), connective tissue growth factor (CTGF), TGF- β1, and fibroblast growth factor (FGF) [4, 6]. In turn, PSCs can also act on PC and inhibit the apoptosis of PC, induce epithelial-mesenchymal transition (EMT), and promote stem cell-like phenotypes in pancreatic cancer cells, resulting in resistance to chemotherapy, distant metastasis and poor prognosis in patients with pancreatic cancer [7–9]. However, the detailed molecular mechanisms underlying the activation of PSCs in pancreatic cancer and the desmoplastic reaction induction of tumor cell proliferation are still unclear. Therefore, understanding the molecular mechanisms that control tumor growth and the desmoplastic reaction in PDAC is important.

EMT is a process defining the progression that cells lose their polarized epithelial character and acquire a migratory mesenchymal phenotype. Consequences of the EMT are the loss of E-cadherin expression and the acquisition of mesenchymal markers including fibronectin or Vimentin [10]. EMT is regulated by a complex network of cytokines, transcription factors, growth factors, signaling pathways, and the tumor microenvironment, exhibiting CSC-like properties. The transition of solid cancer cells from an epithelial to a mesenchymal phenotype enables the cancer cells to gain migratory and invasive properties consequently lead to tumor metastasis and cancer stem cell property [11].

The Hippo/YAP pathway was first discovered by genetic mosaic screens in *Drosophila melanogaster* [12, 13], and since then, increasing evidence has demonstrated that the Hippo pathway also limits organ size in mammalian systems [14, 15] by inhibiting cell proliferation and promoting apoptosis. YES-associated protein (YAP), a main component of the Hippo pathway, has been confirmed to be overexpressed and to participate in the tumorigenesis of a variety of cancers, including breast cancer [16], lung cancer [17], ovarian cancer [18], and liver cancer [19]. Previous studies have demonstrated that YAP-mediated molecular mechanisms in tumors include proliferation and apoptosis through interactions with proteins such as glypican-3 and sox4 as well as the secretion of proteins (such as CTGF and osteopontin) [20, 21], indicating that YAP not only regulates autonomous processes in tumor cells but also may affect the tumor microenvironment. However, little is known regarding YAP expression and its relevance to pathological fibrosis in PDAC.

In this study, we aimed to determine the expression and function of YAP in PDAC and evaluate the relationship between YAP and the desmoplastic reaction in PDAC as well as the underlying molecular mechanisms. Taken together, these results provide additional evidence that YAP contributes to pancreatic cancer progression.

## Methods

### Human tissue specimens and histological analyses

We obtained 72 pancreatic cancer samples and 20 normal pancreatic tissues from the Department of Hepatobiliary Surgery, the First Affiliated Hospital of Xi’an Jiaotong University between 2010 and 2014 after receiving approval from the Ethical Committee of Xi’an Jiaotong University. The pathological TNM status was assessed according to the criteria of the sixth edition of the TNM classification of the American Joint Commission on Cancer (AJCC). The pathological factors were examined by two pathologists. The results are summarized in Table 1. Immunohistochemical staining was performed using a SABC kit (Maxim, Fuzhou, China) according to the manufacturer’s instructions. Briefly, the tissue sections were incubated with primary antibodies overnight at 4 °C and incubated with the appropriate biotinylated secondary antibody for 30 min at room temperature, followed by 30 min of incubation with streptavidin peroxidase (Dako LSAB+HRP kit). After rinsing, the results were visualized using DAB, and the slides were counterstained with hematoxylin. The staining results were scored by 2 pathologists blinded to the clinical data as described previously [22]. The YAP staining status was evaluated according both nucleus and cytoplasm expression. Depending on the percentage of positive cells and staining intensity, YAP staining was classified into four groups: negative (0), weak (1+), moderate (2+) and strong (3+). Specifically, the percentage of positive cells was divided into five grades (percentage scores): < 10% (0), 10–25% (1), 25–50% (2), 50–75% (3), and 75% (4). The intensity of staining was divided into four grades (intensity scores): no staining (0), light brown (1), brown (2), and dark brown (3). YAP staining positivity was determined by the formula: overall scores = percentage score × intensity score. The overall score of ≤3 was defined as negative (0), of > 3 and ≤ 6 as weak (1+); of > 6 and ≤ 9 as moderate (2+), and of > 9 as strong (3+).

**Table 1:** The relationship between expression of YAP and clinical pathological features in pancreatic cancer

	Samples (n)	YAP expression n (%)				
		None	Weak	Moderate	Strong	*P* value^◊^
Sex						0.755
Male	45	8(17.8)	19(42.2)	9(20.0)	9(20.0)	
Female	27	6(22.2)	8(29.6)	6(22.2)	7(26.0)	
Age						0.306
≤55^a^	37	9(24.3)	16(43.2)	6(16.2)	6(16.2)	
>55	35	5(14.3)	11(31.4)	9(25.7)	10(28.6)	
Histological grade						0.780
Well	11	2(18.2)	6(54.5)	1(9.1)	2(18.2)	
Moderate	49	11(22.4)	16(32.7)	11(22.4)	11(22.4)	
Poor	12	1(8.3)	5(41.7)	3(25.0)	3(25.0)	
TNM stage (AJCC)						0.012*
I	2	1(50.0)	1(50.0)	0(0)	0(0)	
II	59	12(20.3)	26(44.1)	9(15.3)	12(20.3)	
III	6	1(16.7)	0(0)	4(66.7)	1(16.7)	
IV	5	0(0)	0(0)	2(40.0)	3(60.0)	
pT status						0.135
T1	0	0(0)	0(0)	0(0)	0(0)	
T2	8	1(12.5)	3(37.5)	2(25)	2(25)	
T3	58	12(20.7)	24(41.4)	9(15.5)	13(22.4)	
T4	6	1(16.7)	0(0)	4(66.7)	1(16.7)	
pN status						0.755
N0	55	11(20.0)	22(40.0)	10(18.2)	12(21.8)	
N1	17	3(17.7)	5(29.4)	5(29.4)	4(23.5)	
pM status						0.028*
M0	67	14(20.9)	27(40.3)	13(19.4)	13(19.4)	
M1	5	0(0)	0(0)	2(40.0)	3(60.0)	

^◊^χ^2^ test; *Significant different, *P*< 0.05

^a^Median age

### Cell lines, culture conditions and reagents

Human pancreatic cancer cell lines (AsPC-1, BxPC-3, CFPAC-1, Panc-1, and SW-1990) were purchased from the Chinese Academy of Sciences Cell Bank of Type Culture Collection (CBTCCCAS, Shanghai, China). According to the instructions, all cell lines were cultured in the proper medium (HyClone, Logan, USA) supplemented with 10% fetal bovine serum (FBS), 100 U/ml penicillin and 100 μg/ml streptomycin. The cultures were incubated at 37 °C in a humidified atmosphere containing 5% CO_2_. Recombinant human TGF-β1 was purchased from PeproTech (Rocky Hill, USA). Detailed information regarding the antibodies used in this study is presented in Additional file 1: Table S1. All reagents were stored as recommended by the manufacturer.

### Genetically engineered transgenic mice

Pdx1-Cre mice, LSL-Kras^G12D^ mice and Trp53^fl/fl^ mice were purchased from the Nanjing Biomedical Research Institute of Nanjing University, Nanjing, China. The breeding of LSL-Kras^G12D/+^; Pdx1-Cre (KC) transgenic mice was achieved by crossing LSL-Kras^G12D^ mice with Pdx1-Cre mice. LSL-Kras^G12D/+^; Trp53^fl/+^; Pdx1-Cre (KPC) mice were obtained by firstly crossing Trp53^fl/fl^ mice with Pdx1-Cre mice to generate Trp53^fl/fl^; Pdx1-Cre offspring. Trp53^fl/fl^; Pdx1-Cre mice were then crossed with LSL-Kras^G12D^ mice to generate KPC animals. All mice were housed under pathogen-free conditions and with free access to water and food. All experimental protocols were approved by the Ethical Committee of the First Affiliated Hospital of Medical College, Xi’an Jiaotong University, Xi’an, China.

### Stable YAP shRNA lentiviral transfection

YAP shRNA (shYAP) and negative control shRNA (shNC) in eukaryotic GV248 lentiviral vectors were purchased from GeneChem Co, Ltd. (Shanghai, China). The target sequence for YAP shRNA was CACCAAGCTAGATAAAGAA, and the negative control sequence was TTCTCCGAACGTGTCACGT. Cells were seeded at 1 × 10^5^ cells/well into 6-well plates 24 h prior to transfection. Transfection was carried out using lentiviral particles (Pan-1 MOI = 10; BxPC-3 MOI = 20), polybrene (5 μg/ml) and ENi.S according to the manufacturer’s protocol. Then, 12 h post-transfection, virus-containing medium was replaced with complete medium, and 96 h post-transfection, all cells were selected with puromycin (Merck, USA) at a final concentration of 5 μg/ml (Panc-1) or 4 μg/ml (BxPC-3) for 10 days. Cells were then maintained in 2.5 μg/ml (Panc-1) or 2 μg/ml (BxPC-3) puromycin. For the generation of stable transfected cells, media was changed three times a week. After 3 weeks, puromycin-resistant colonies were isolated for further study. The stable YAP-suppressed PCs and the Control PCs were named sh-YAP and sh-NC, respectively. The effect of gene silencing was evaluated by qRT-PCR and western blot.

### Immunofluorescence

For fluorescent immunocytochemistry, the pancreatic cancer cells and pancreatic stellate cells were fixed for 20 min in 4% paraformaldehyde in PBS, and the endogenous peroxidase activity was quenched with 3% hydrogen peroxide. The specimens were permeabilized with 0.3% Triton X-100 in PBS for 15 min on ice, pre-blocked for 60 min with bovine serum albumin (BSA) at room temperature, and incubated with primary antibody overnight at 4 °C. Staining was detected with the corresponding fluorescein-conjugated secondary antibodies (Jackson ImmunoResearch). Slides were mounted and examined using a Zeiss Instruments confocal microscope.

### Colony formation assay

Cells (1000 shYAP or shNC cells) were seeded into a 35-mm petri dish and allowed to adhere overnight. Cells were further cultured for 2 weeks to allow colonies to form. At the indicated time point, colonies were fixed with 4% paraformaldehyde, stained with 0.1% crystal violet solution, rinsed and then imaged. The colonies > 0.5 mm in diameter were counted using a microscope (Nikon Eclipse Ti-S, Japan) at a magnification of 400 × .

### Cell viability assay

Cell viability was measured using an MTT (3-(4,5-dimethylthiazol-2-yl)-2,5-diphenyltetrazolium bromide) assay (Biotime). The cells were seeded into 96-well plates at a density of 5000 cells per well and incubated overnight in 10% FBS medium. After incubation for 24, 48, and 72 h at 37 °C and 5% CO_2_, the cell proliferation rate was determined. Briefly, 20 μl of MTT solution (5 mg/ml in distilled water) was added to each well, and the cells were incubated for 4 h at 37 °C, after which the medium was removed. Then, 150 μl of DMSO was added, and the optical density (OD) was measured at 490 nm on a multifunctional microplate reader (POLARstar OPTIMA; BMG, Offenburg, Germany).

### Real-time PCR assay

Total RNA was extracted using a Fastgen1000 RNA isolation system (Fastgen, Shanghai, China) according to the manufacturer’s protocol. Total RNA was reverse-transcribed into cDNA using a Prime Script RT reagent kit (TaKaRa, Dalian, China). Real-time PCR was used to quantitatively examine the expression of YAP, CTGF, E-cadherin, N-cadherin and Vimentin at the mRNA level. Real-time PCR was conducted according to a previous report [23]. The PCR primer sequences for YAP, CTGF, E-cadherin, N-cadherin, Vimentin and β-actin are shown in Additional file 1: Table S2. The expression level of each target gene was determined using β-actin as the normalization control. Relative gene expression was calculated using the 2^-ΔΔCt^ method [24].

### Enzyme-linked immunosorbent assay (ELISA)

Cells were conditioned in serum-free medium for 48 h. The culture media were collected and centrifuged at 1500 rpm for 5 min to remove particles, and the supernatants were frozen at − 80 °C until use. The production of TGF-β1 in the supernatants of PSCs was assessed by ELISA using a commercially available ELISA kit (R&D Systems, USA) according to the manufacturer’s recommendations.

### Cell invasion assay

Transwell chambers (pore size, 8.0 μm; Millipore, Billerica, USA) were coated with Matrigel (BD Biosciences, Oxford, UK). PCs were cultured in 6-well plates in medium containing 1% FBS for 24 h before drug treatment. PCs (200 μl, 5 × 10^4^) suspended in DMEM containing 1% FBS were seeded in the top chamber, and 500 μl of medium containing 10% FBS was placed in the lower chamber as a chemoattractant. The Transwell chamber was incubated for 48 h. The invaded cells on the bottom surface of the filter were fixed with methanol and stained with crystal violet (Boster Biological Technology Ltd., Wuhan, China). Cell migration and invasion were determined by counting the stained cells under a light microscope in 10 randomly selected fields.

### Western blot analysis

Total protein was extracted using RIPA Lysis Buffer (Beyotime, Guangzhou, China), and the protein concentration was determined using a BCA protein assay kit (Pierce, Rockford, USA) according to the manufacturer’s instructions. Then, a western blot assay was performed as previously described. The primary antibodies used are listed in Additional file 1: Table S1. The protein expression was visualized by enhanced chemiluminescence (Millipore, USA). Images were captured using a ChemiDoc XRS imaging system (Bio-Rad, USA), and Quantity One image software was used for the densitometry analysis of each band; β-actin was used as an internal loading control.

### Isolation and culture of human pancreatic stellate cells

Normal pancreatic tissues (1.0–1.5 g) obtained from patients undergoing a pancreatic partial resection for benign pancreatic conditions at the First Affiliated Hospital of Xi’an Jiaotong University were immediately collected in sterile ice-cold Hanks balanced salt solution (HBSS) containing 100 U/ml penicillin and 100 μg/ml streptomycin (Gibco). The histological diagnostic assessment of specimens was confirmed by pathologists. Human pancreatic stellate cells (PSCs) were isolated using a density gradient method as previously described. Isolated PSCs were maintained at 37 °C with 5% CO_2_ in DMEM/F12 (HyClone, Logan, USA) medium supplemented with 10% heat-inactivated fetal bovine serum (FBS) (HyClone), 100 U/ml penicillin and 100 μg/ml streptomycin. PSCs were identified by oil red staining of intracellular fat droplets and immunofluorescence of α-smooth muscle actin (α-SMA). Cells cultured in the above medium conditions for 24 h were used in additional experiments.

### PC-PSC co-culture models

After pancreatic cancer cells were cultured in media supplemented with 10% FBS and grown to 50% confluence, the media was changed to contain 1% FBS, 100 U/ml penicillin and 100 μg/ml streptomycin. Two days later, cancer cell conditioned medium was collected, centrifuged and filtered prior to incubation with isolated PSCs as previously described [3], and the PSCs were incubated with the conditioned medium for up to 2 days. For the direct PC-PSC co-culture model, PCs and PSCs were proportionally mixed (cell proportion, 2:1) and seeded into 6-well plates.

### In vivo tumor model

Mice were housed and maintained under specific pathogen-free conditions in facilities approved by the Animal Care and Use Committee guidelines of the Xi’an Jiaotong University, Shaanxi, China. Investigations were conducted in accordance with ethical standards, the Declaration of Helsinki and national and international guidelines and were approved by the authors’ institutional review board. The mice were used according to institutional guidelines when they were 6 to 8 weeks of age. Cells were resuspended in a 1:1 (*v*/v) mixture of culture medium and Matrigel (BD Biosciences, San Jose, CA, USA), and 1 × 10^6^ BxPC-3-shNC cells, 1 × 10^6^ BxPC-3-shYAP cells, 0.8 × 10^6^ BxPC-3-shNC cells + 2 × 10^5^ PSCs, or 0.8 × 10^6^ BxPC-3-shYAP cells + 2 × 10^5^ PSCs were injected s.c. into the right flank of nude mice. A total of 5 mice per group were used. After 8 weeks, the animals were sacrificed, and the subcutaneous tumors were isolated. Tumors were fixed in formalin as soon as possible and embedded in paraffin. Tumor volume was calculated as (length/2) × (width^2). Tumor samples were analyzed using H&E staining. Representative images were taken of each tumor using a light microscope at × 400 magnification.

### Statistical analysis

Statistical analysis was performed using the SPSS statistical software package (version 13.0). The significance of the patient specimen data was determined using Pearson’s correlation coefficient or Fisher’s exact test. The significance of the in vitro and in vivo data was determined using Student’s t-test (2-tailed), Mann–Whitney test (2-tailed), or one-way ANOVA. *P* < 0.05 was considered statistically significant.

## Results

### YAP is overexpressed in pancreatic cancer tissues

**Fig. 1:**
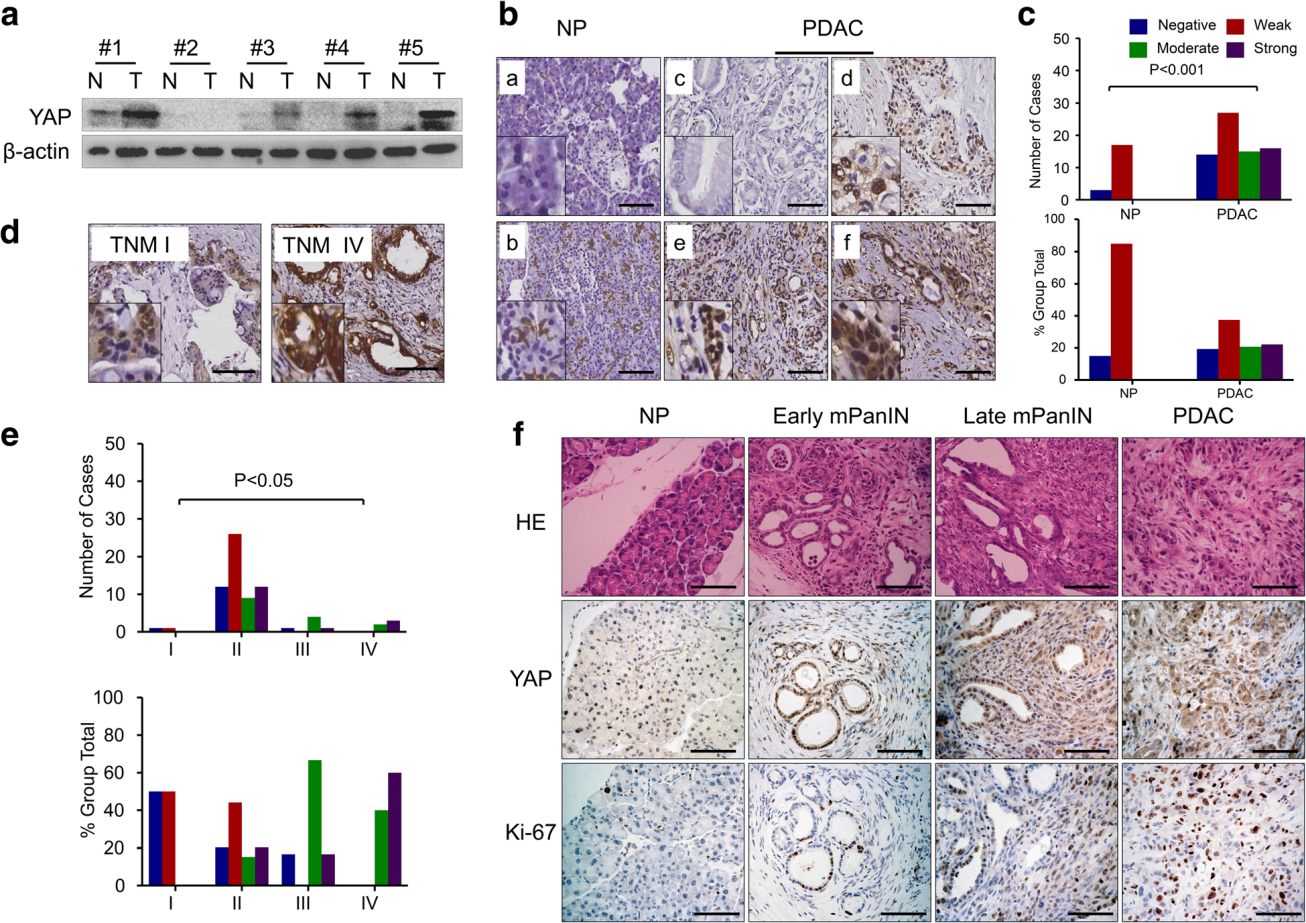
Expression of YAP and its association with clinical pathological features of PDAC. (**A**) Representative western blot analyses showing YAP expression levels in PDAC and NP tissues. (**B**) Representative IHC images of normal pancreatic tissue with negative (a) and weak (b) YAP staining, and PDAC with negative (c), weak (d), moderate (e) and strong (f) YAP staining are shown (bar, 100 μm). (**C**) YAP expression was markedly increased in PDAC compared with NP tissues (*P* < 0.001). (**D**) Representative IHC images showing YAP expression levels in PDAC with TNM I and TNM IV (bar, 100 μm). (**E**) Positive association of YAP expression with TNM stage in PDACs (*P* < 0.05). (**F**) Representative hematoxylin and eosin (HE) staining and immunostaining for YAP and Ki-67 in normal pancreatic tissues (NP) from wild-type (WT) mouse, early and late mPanIN pancreatic tissues from LSL-Kras^G12D/+^; Pdx1-Cre (KC) mouse model and PDAC tissues from LSL-Kras^G12D/+^; Trp53^fl/+^; Pdx1-Cre (KPC) mouse model (bar, 100 μm)

To determine whether YAP is overexpressed at the protein level in human PDAC tissues, we examined YAP expression in five human pancreatic cancer tissues and the corresponding normal pancreatic tissue via western blot. The results show that the levels of YAP protein in pancreatic cancer tissues were significantly elevated compared with normal pancreatic tissues (Fig. 1A). To further confirm these results, pancreatic tissue sections from 72 patients identified as PDAC and 20 cases of normal pancreatic specimens were analyzed using immunohistochemistry (IHC). Intense IHC staining of YAP was detected in the cytoplasm and nucleus of cancer cells, but cells exhibited a predominantly nuclear localization pattern, whereas rare staining events were observed in normal pancreatic tissues (Fig. 1B). The statistics for YAP expression levels in different pancreatic tissue groups are shown in Table 1. Figure 1 shows representative pictures of negative (0; Fig. 1Bc), weak (1+; Fig. 1B d), moderate (2+; Fig. 1B e) and strong (3+; Fig. 1Bf) YAP staining in pancreatic cancer. As shown in Fig. 1B, no (Fig. 1Ba) or moderate (Fig. 1Bb) YAP immunoreactivity was observed in normal pancreatic tissues. YAP expression was significantly increased in PDAC compared to normal pancreatic tissues (*P* < 0.001; Fig. 1C). Notably, the χ2 analysis revealed that histologic markers of aggressive disease, including tumor–node–metastasis (TNM) stage (*P* = 0.010; Fig. 1D and E), and pM stage (*P* = 0.038) were significantly associated with YAP expression levels (Table 1). LSL-Kras^G12D/+^; Pdx1-Cre (KC) and LSL-Kras^G12D/+^; Trp53^fl/+^; Pdx1-Cre (KPC) mice, in which Pdx1 induces the expression of mutant Kras alone or together with mutant Trp53 in murine pancreatic epithelium, fully recapitulate the pathogenesis of human PDAC and are generally regarded as two of the best genetically engineered mouse models (GEMMs) for human PDAC. Next, we detected the YAP expression in KC and KPC mice, and we found that YAP protein abundance was also markedly greater in pancreatic tissue from KC mice that had early and late pancreatic intraepithelial neoplasia (PanIN) or KPC mice that had fully established PDAC compared with wild-type mice (Fig. 1F). These findings indicated that YAP plays critical roles in PDAC development and progression and may be a valuable biomarker for this disease.

### Knockdown of YAP inhibits pancreatic cancer cell proliferation in vitro

**Fig. 2:**
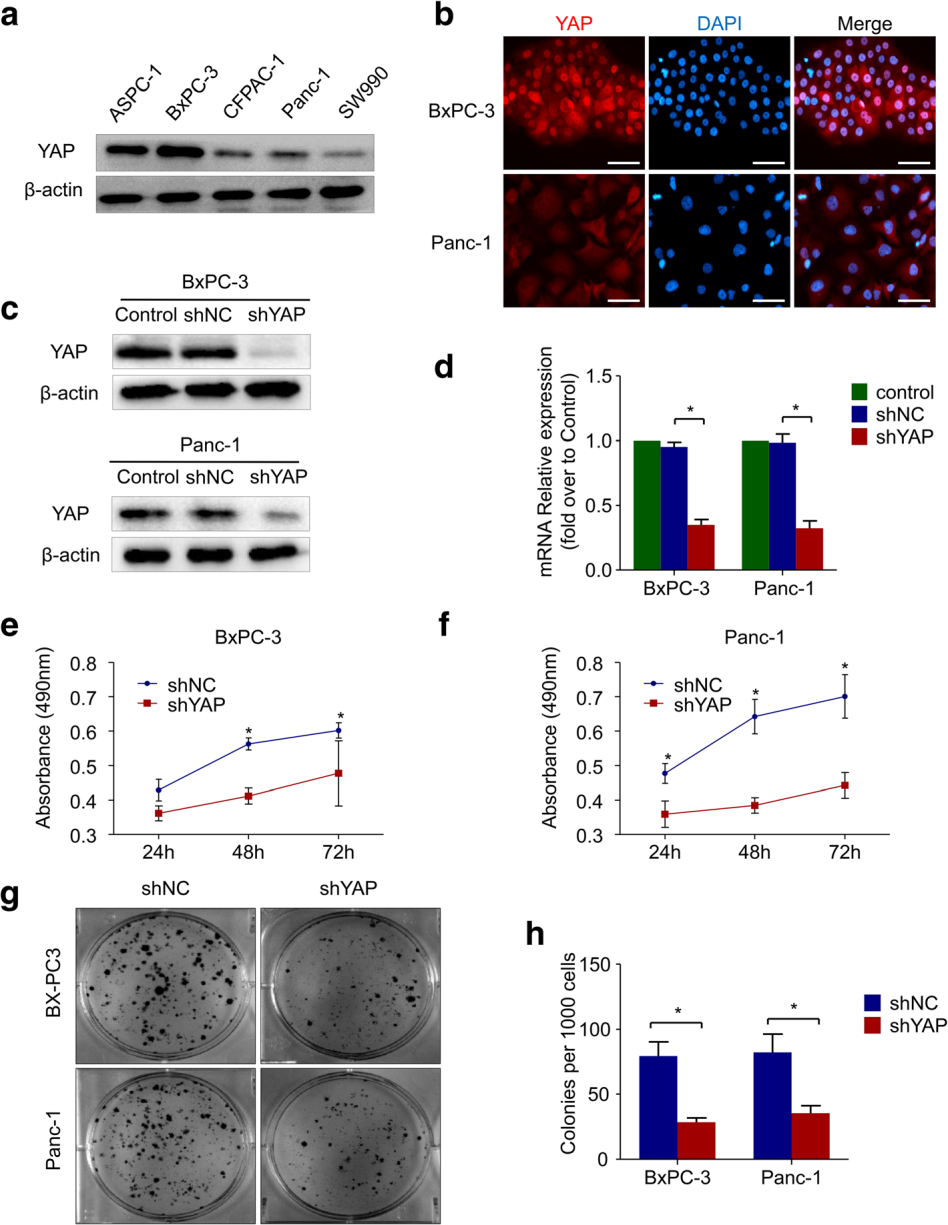
Knockdown of YAP inhibits pancreatic cancer cell proliferation. **a** Western blot showing expression of YAP protein in PDAC cell lines. **b** Immunofluorescence indicating that YAP (red) was expressed in both the cytoplasm and nucleus but was mainly located in the nucleus. Nuclei were stained with DAPI (blue) (bar, 50 μm). After transfection of Panc-1 and BxPC-3 cells with YAP-shRNA and Negative control lentiviral vectors, **c** The expression level of YAP protein was determined by western blot, which showed that the YAP protein level was significantly reduced. **d** the YAP mRNA expression level was determined using real-time PCR and normalized to that of β-actin, and YAP mRNA expression was clearly suppressed by YAP-shRNA. **e** & **f** BxPC-3 and Panc-1 cells were transfected with YAP-shRNA lentiviral vectors and Negative control lentiviral vectors. At the indicated time points (24, 48 and 72 h), cell viability was assessed using MTT assays. **g** & **h** The effects of YAP-shRNA on the colony forming ability of Panc-1 and BxPC-3 cells. All of the data are presented as the mean ± SD of three independent experiments. Column: mean; bar: SD; *P < 0.05

To determine the effect of YAP on PDAC growth, we analyzed its expression in PDAC cell lines. YAP was highly expressed in AsPC-1, BxPC-3 and Panc-1 cells but expressed at relatively low levels in CFPAC-1 and SW-1990 cells (Fig. 2a). Immunofluorescence showed that YAP was located in the nucleus and the cytoplasm of the BxPC-3 and Panc-1 cells (Fig. 2b). Accordingly, we defined BxPC-3 and Panc-1 cells as samples with differential expression for further experiments. The lentiviral vector YAP-shRNA was used to suppress YAP expression in two pancreatic cancer cell lines, BxPC-3 and Panc-1. From qRT-PCR and western blot results, we found that the YAP gene was significantly knocked down after YAP-shRNA transfection (Fig. 2c and d). At various time points (24, 48, and 72 h), the proliferation rate of BxPC-3-shYAP and Panc-1-shYAP cells were determined with an MTT assay. The results show that YAP knockdown inhibited the proliferation of both pancreatic cancer cell lines (Fig. 2e and f). Next, we detected the effect of YAP on the clone formation capability of Panc-1 and BxPC-3 cells, and the colony formation ability of both cell lines was clearly decreased after knockdown of YAP expression (Fig. 2g and h).

### Knockdown of YAP inhibits pancreatic cancer cell invasion through inhibiting EMT

**Fig. 3:**
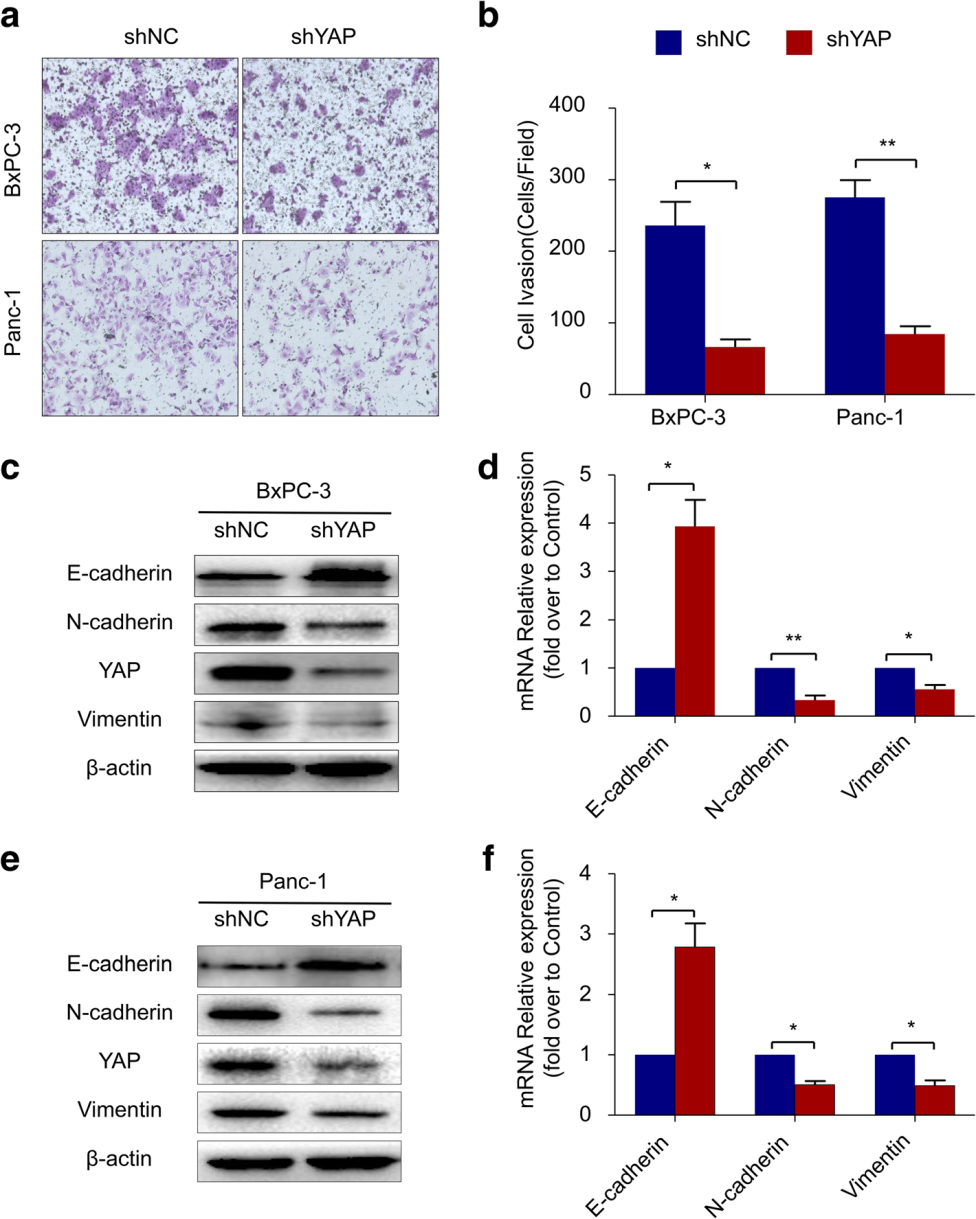
Knockdown of YAP inhibits pancreatic cancer cell invasion through inhibiting EMT **a** Transwell chamber assays of BxPC-3 and Panc-1 cells. The pretreated cells were seeded into a Matrigel-coated invasion chamber for an additional 48 h. **b** The invasive cells were quantified by counting the number of cells in 10 random fields at 100 × magnification. **c** & **e** Western blot assays were performed to evaluate the effect of YAP-shRNA on the protein expression of E-cadherin, N-cadherin, Vimentin and YAP in BxPC-3 and Panc-1 cells. **d** & **f** Real-time PCR was performed to evaluate the effect of YAP-shRNA on the mRNA expression of E-cadherin, N-cadherin and Vimentin. All of the data are presented as the mean ± SD of three independent experiments. Column: mean; bar: SD; *P < 0.05; **P < 0.01

To elucidate the role of YAP in the invasive ability of pancreatic cancer cells, we knocked down YAP expression with the lentiviral vector YAP-shRNA in BxPC-3 cells and Panc-1 cells. Using Transwell chamber assays with Matrigel, a significant decrease in the invasion of shYAP cells was observed compared to shNC cells (Fig. 3a and b). Furthermore, western blot verified that knockdown of YAP resulted in a marked decrease in the expression of N-cadherin and Vimentin and a significant increase in E-cadherin (Fig. 3c and e), consistent with reversion to an epithelial phenotype. These observations were confirmed at the mRNA level using real-time PCR (Fig. 3d and f). Together, these data suggested that YAP might participate in the invasion process of PDAC by regulating EMT phenotypes.

### Overexpression of YAP is associated with desmoplastic reaction via activation of pancreatic stellate cells

**Fig. 4:**
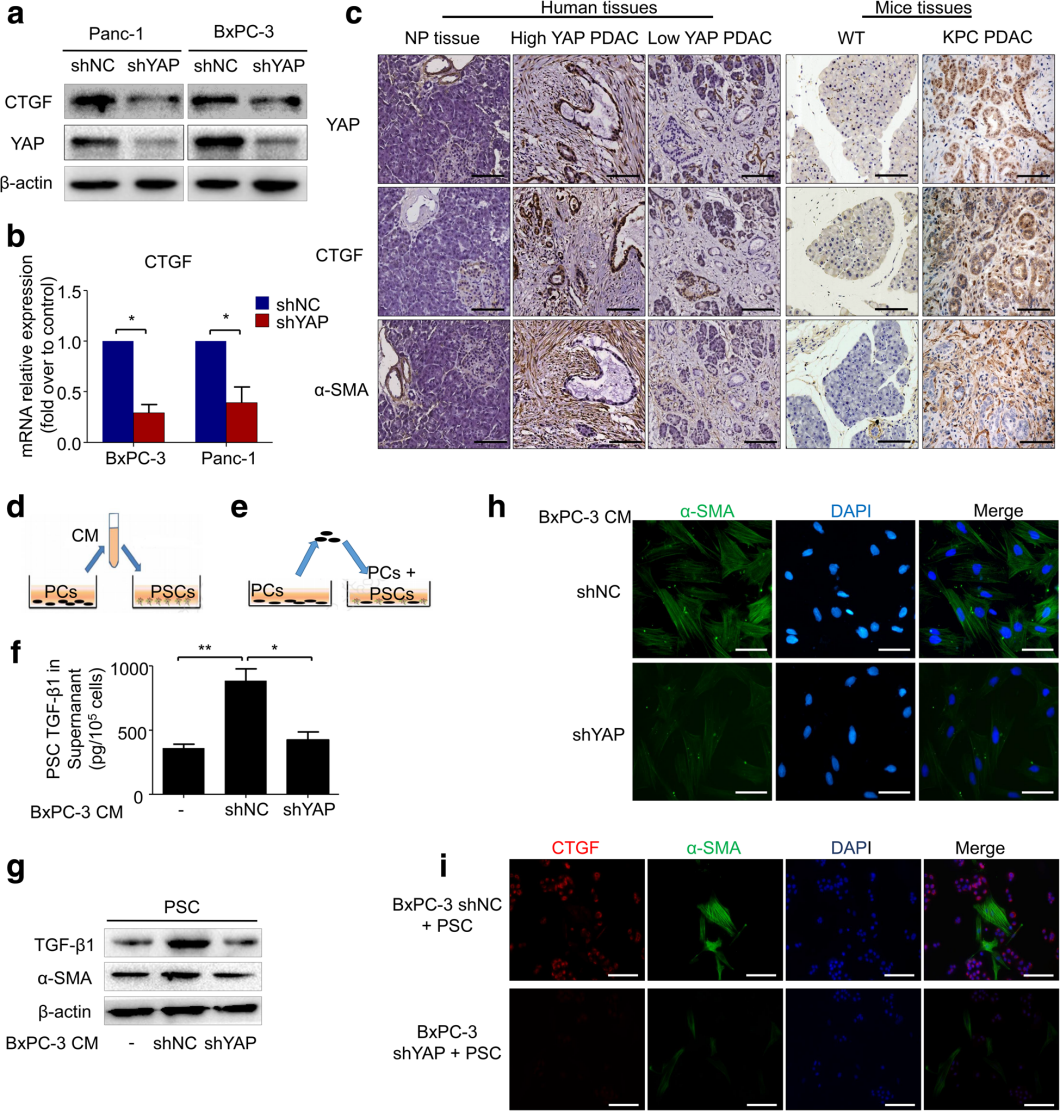
Knockdown of YAP expression suppresses cancer cell-induced PSC activation. Western blot (**a**) and Real-time PCR assays (**b**) were performed to evaluate the effect of YAP-shRNA on the expression of CTGF at the mRNA and protein levels in BxPC-3 and Panc-1 cells. **c** The representative IHC staining for YAP, CTGF and α-SMA in the NP and PDAC specimens, accompanied by strong YAP staining or with weak YAP staining and NP specimens from WT mouse and PDAC specimens from a KPC mouse model (bar, 100 μm). **d** A schematic diagram of indirect co-culture conditions. **e** A schematic diagram of direct co-culture conditions. **f** PSCs were treated with mixed conditioned medium from BxPC3-shNC or BxPC-3-shYAP cells for 48 h; then, the supernatant of PSCs was collected, and an ELISA was performed to evaluate the TGF-β1 level. **g** PSCs were treated with mixed conditioned medium from BxPC3-shNC or BxPC-3-shYAP cells for 48 h, and western blot assays were performed to evaluate the α-SMA and TGF-β1 levels. **h** PSCs were treated with mixed conditioned medium from BxPC3-shNC or BxPC-3-shYAP cells for 48 h, and cells were stained with an anti-α-SMA antibody (green) and counterstained with DAPI (blue) to identify nuclei. Representative images demonstrate that the α-SMA level of PSCs in conditioned medium from BxPC-3-shYAP cells was reduced significantly (bar, 50 μm). **i** PCs (BxPC-3-shYAP or BxPC3-shNC) and PSCs were cultured together for 48 h. Then, immunofluorescence analysis was performed to detect α-SMA (green) and CTGF (red) expression in the cells (bar, 100 μm). *P < 0.05

Several studies have indicated that CTGF is a target of YAP. Consistent with our results, real-time PCR and western blot showed that CTGF was down-regulated after YAP knockdown (Fig. 4a and b). The IHC results also showed a positive correlation between YAP and CTGF staining in pancreatic cancer (Fig. 4c). We noted that both pancreatic cancer cells and pancreatic satellite cells stained positive for YAP, and YAP could be found in both the nucleus and the cytoplasm (Fig. 4c). YAP expression was positively correlated with α-SMA expression, a marker for activated stellate cells, and this correlation was also present in the *KPC* PDAC tissues (Fig. 4c). Next, we further investigated the effects of YAP on the tumor-stroma interactions between PCs and PSCs, and PC-PSC co-culture models were designed. The BxPC-3 has the highest YAP expression and this cell line was derived from the primary pancreatic cancer. So, we choose BxPc-3 for the further investigation of the interaction among YAP, PCs and PSCs. For the indirect co-culture model (Fig. 4d), PC supernatant was prepared after BxPC-3-shNC or BxPC-3-shYAP cells were cultured in conditioned medium (CM) for 24 h. Then, the conditioned medium mixture was added to the serum-free starvation-synchronized PSCs. After 48 h, PSC activation was determined by examining the α-SMA level using immunofluorescence labeling and western blot. As shown in Fig. 4g and h, the PSC activation level was reduced after incubation with BxPC-3-shYAP-CM compared with BxPC-3-shNC-CM, as revealed by α-SMA expression. Increased TGF-β1 synthesis and secretion is a hallmark of activated PSCs. The immunoblotting and ELISA results confirmed the inhibitory effect of BxPC-3-shYAP-CM on PSC activation (Fig. 4f and g). To investigate the effects of YAP on the activation of PSCs in a PC-PSC co-culture system (Fig. 4e), immunofluorescence was used to visualize the CTGF and PSC activation in the co-culture system. As shown in Fig. 4i, the immunofluorescence results indicate that the PSC activation level was reduced in indirect co-culture conditions after YAP knockdown in BxPC-3 cells, as revealed by α-SMA staining. Surprisingly, CTGF was mainly present in the PCs and was reduced after YAP knockdown in BxPC-3 cells, as revealed by CTGF staining with α-SMA that marked PSCs. Taken together, these data indicated that down-regulation of YAP expression in PCs inhibited PSC activation in a co-culture system, and this may be associated with a decrease in CTGF.

### YAP is a critical mediator of TGF-β1/SMAD2-induced EMT and cell invasion

**Fig. 5:**
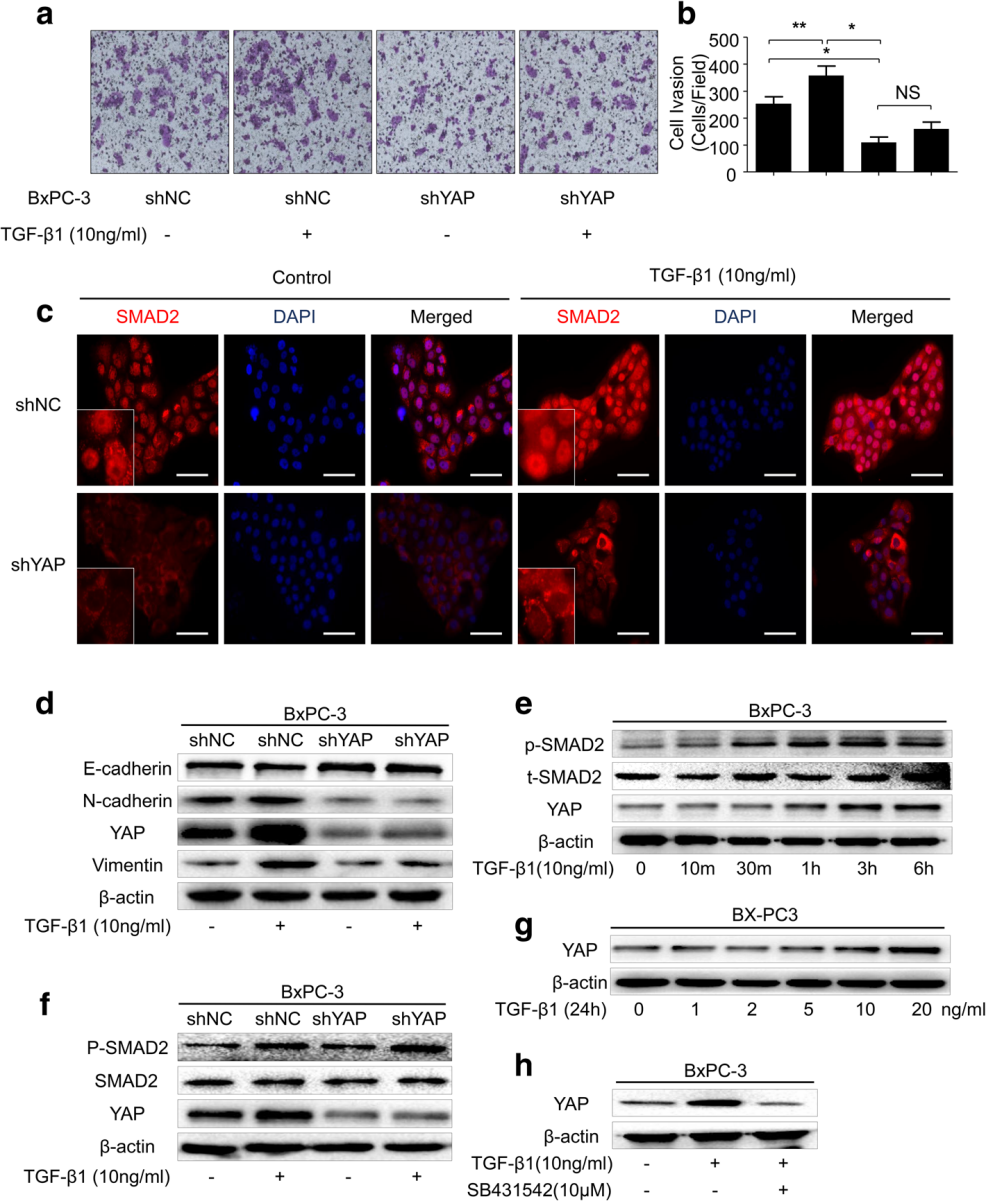
YAP is a critical mediator of TGF-β1/SMAD2-induced EMT and cell invasion. **a** The effect of TGF-β1 (10 ng/ml) on BxPC-3-shYAP and BxPC-3-shNC PC invasion capability was assessed using a Matrigel invasion assay. PCs with or without TGF-β1 (10 ng/ml) pretreatment for 48 h were seeded into Matrigel-coated invasion chambers. **b** The invasive cells were quantified by counting the number of cells in 10 random fields at 100 × magnification. **c** BxPC-3-shYAP and BxPC-3-shNC PCs were pretreated with or without TGF-β1 (10 ng/ml) for 6 h, and cells were stained with an anti-Smad2 antibody (red) and counterstained with DAPI (blue) to identify nuclei (bar, 50 μm). Representative images demonstrate that knockdown of YAP not only reduced TGF-β1-induced nuclear accumulation of Smad2 but also diminished whole–cell Smad2 levels. **d** BxPC-3-shYAP and BxPC-3-shNC PCs were pretreated with or without TGF-β1 (10 ng/ml) for 48 h, and then, western blot assays were performed to evaluate the expression of E-cadherin, N-cadherin, Vimentin and YAP at the protein level. **e** BxPC-3 cells were treated with TGF-β1 (10 ng/ml) for the indicated time (10 min, 30 min, 1 h, 3 h and 6 h), and western blot assays were performed to evaluate the expression of YAP and t-SMAD2 and the p-SMAD2 level. **f** BxPC-3-shYAP and BxPC-3-shNC PCs were pretreated with or without TGF-β1 (10 ng/ml) for 1 h, and then, western blot assays were performed to evaluate the p-SMAD2 and YAP level. **g** BxPC-3 cells were treated with TGF-β1 at the indicated concentration (1, 2, 5, 10 and 20 ng/ml) for 24 h, and western blot assays were performed to evaluate the protein expression of YAP. **h** BxPC-3 cells were pretreated with the specific TGF-β1 inhibitor SB431542 (10 μM) for 1 h and then treated with TGF-β1 (10 ng/ml) for 24 h, and western blot assays were performed to evaluate the protein expression of YAP. Column: mean; bar: SD; *P < 0.05; **P < 0.01

PSC activation has been recognized as a major driving force in the tumor microenvironment that promotes pancreatic cancer progression, and TGF-β1 plays an important role in tumor-stroma interactions. Therefore, we continued to elucidate whether TGF-β1 could reverse the YAP inhibition of pancreatic cancer invasion and EMT phenotypes. BxPC-3 cells were cultured for 48 h with or without TGF-β1. The results showed that the invasion ability (Fig. 5a and b) and the mesenchymal-related gene (N-cadherin and Vimentin) expression in BxPC-3-shNC cells were significantly increased after treatment with 10 ng/ml TGF-β1. However, simultaneous knockdown of YAP using shRNA abolished TGF-β1-induced cell invasion (Fig. 5a and b) and EMT (Fig. 5d). Studies have indicated that YAP can bind TGF-β1-activated SMAD complexes to control SMAD localization and activity in a variety of cell types, including mammary epithelial cells [25] and breast cancer cells [26]. The results showed that simultaneous knockdown of YAP using shRNA did not affect the p-SMAD2 level (Fig. 5f). However, the immunofluorescence results showed that SMAD2 nuclear location was significantly decreased when YAP was knocked down (Fig. 5c), and it also abolished TGF-β1-induced SMAD2 nuclear localization (Fig. 5c). We also found that YAP was significantly up-regulated after treatment with TGF-β1 in a time-dependent and dose-dependent manner (Fig. 5e and g), and this up-regulation was reversed after treatment with the TGF-β1 receptor inhibitor SB431542 (Fig. 5h). Taken together, our observations indicate that YAP is a critical mediator of TGF-β1-induced tumorigenic events, including EMT and cell invasion.

### Knockdown of YAP in pancreatic cancer cells suppresses the tumor growth and desmoplastic reaction in vivo

**Fig. 6:**
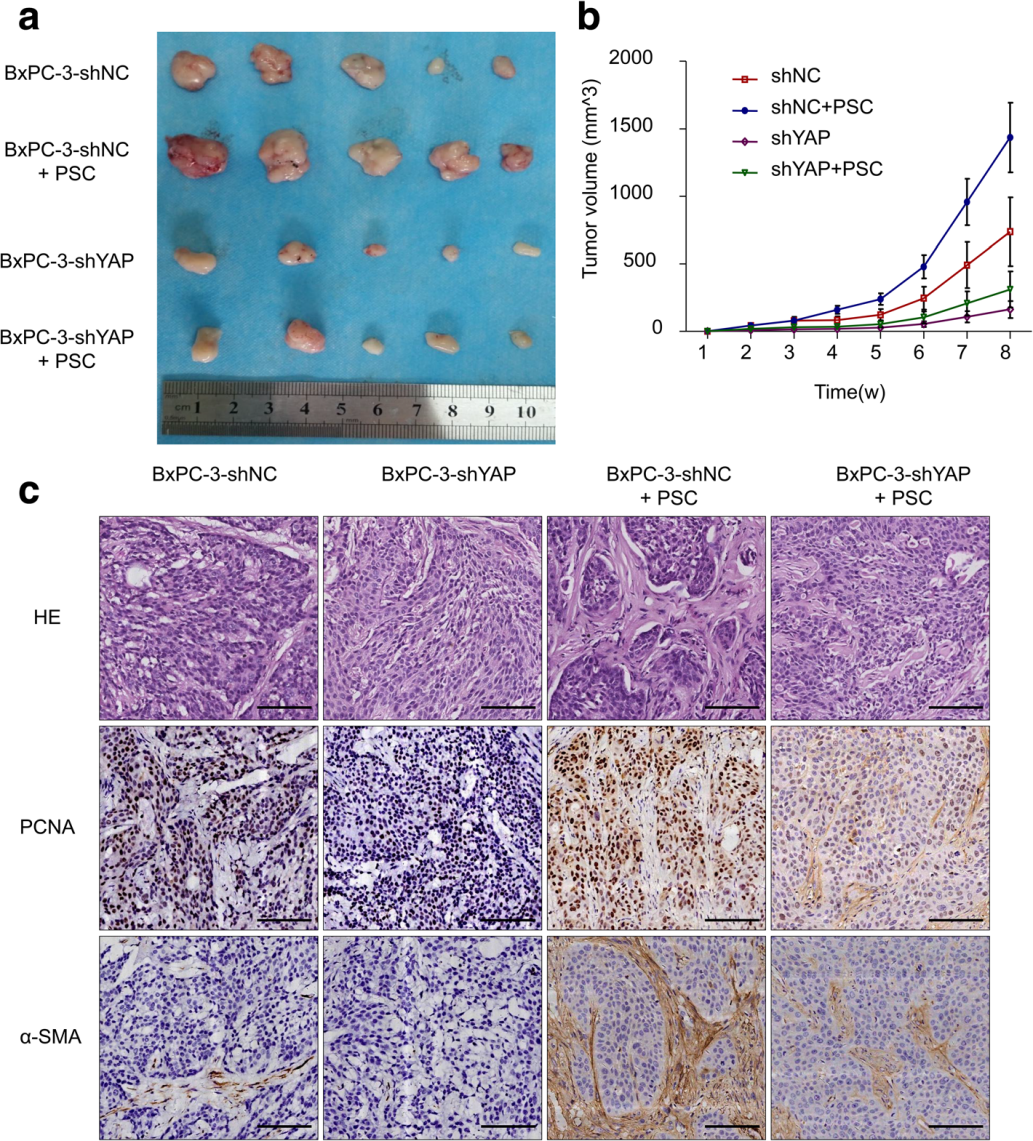
Knockdown of YAP in PCs suppresses tumor growth and desmoplastic reaction in vivo. **a** Cell mixtures containing BxPC-3-shNC, BxPC-3-shYAP, BxPC-3-shNC+PSCs and BxPC-3-shYAP+PSCs were implanted subcutaneously into the flank of BALB/c nude mice. Representative photographs of BxPC-3-shNC, BxPC-3-shYAP, BxPC-3-shNC+PSC, and BxPC-3-shYAP+PSC co-implantation mice bearing xenograft tumors. **b** Tumor volumes were determined by measuring the width and length of the tumors every week. Mean (*n* = 5); bars, SD. **c** Representative images of hematoxylin and eosin (HE staining and immunostaining for PCNA and α-SMA in tumor tissues from mice subcutaneously injected with pancreatic cancer cells (bar, 100 μm)

Based on the promising in vitro findings reported above, we next sought to test the role of YAP in pancreatic cancer cells on tumor progression and PSC activation in vivo. We established a subcutaneous pancreatic cancer xenograft model in nude mice through injection of PCs alone or PCs plus PSCs. The tumor volume was monitored, as shown in Fig. 6a, and we noted that BxPC-3-shYAP cells exhibited a notable reduction in tumor growth rates compared to BxPC-3-shNC cells. Moreover, co-injection of BxPC-3-shNC cells and PSCs resulted in a significant increase in tumor growth rates compared to BxPC-3-shNC cells alone. There was a significant induction of tumor growth when PSCs were co-injected with BxPC-3-shYAP cells in which the YAP expression was repressed (Fig. 6a and b). At the same time, we observed different histologic structures, especially in the stromal component, in the tumor tissues. The results showed that there were more stromal components in the BxPC-3-shNC+PSC co-injection group compared to the BxPC-3-shYAP+PSC co-injection group. Consistent with the in vitro studies, the IHC results showed that the proliferation of cancer cells was inhibited in the BxPC-3-shYAP group, as there was less and weaker expression of PCNA in the BxPC-3-shYAP group compared with the BxPC-3-shNC group (Fig. 6c). Moreover, the tumor tissues from mice in the BxPC-3-shYAP+PSC co-injection group exhibited lower staining levels of the stromal marker α-SMA compared with the BxPC-3-shNC+PSC co-injection group (Fig. 6c). Taken together, our in vivo findings indicate that YAP is critical in tumor growth and the desmoplastic reaction.

## Discussion

PDAC is a notoriously aggressive malignancy that responds poorly to most chemotherapeutic agents, and the major reason is the complex pancreatic cancer tumor-microenvironment, which contributes to tumor invasion and the chemotherapy-resistant phenotype of pancreatic cancer cells. Previous studies have demonstrated that YAP, which is the main effector of the Hippo pathway, is an attractive target of investigation in mammalian malignancies, and it has been found to play an important role in breast cancer [16], lung cancer [17], ovarian cancer [18], and liver cancer [19] development. In this study, we explored the role of YAP in the invasiveness and proliferation of pancreatic cancer cells and the activation of PSCs.

Our study showed that YAP expression was highly up-regulated in pancreatic cancer tissues compared to normal pancreatic tissues. Moreover, the YAP expression level was well correlated with the TNM stage of pancreatic cancer patients. Furthermore, our results showed that YAP expression was generally more intense in the nucleus than in the cytoplasm, indicating that YAP was in an active phenotype and likely promoting pancreatic cancer progression. Consistent with the previous studies [27, 28], our study showed that the staining of YAP in normal pancreatic tissues is restricted to acinar cells and small ducts, subpopulations that likely represent the normal function of YAP in maintaining tissue homeostasis. Furthermore, YAP is also up-regulated in mouse PanIN and as well as in stellate cells associated with PDAC. These findings indicated that YAP may be involved in pancreatic tissue regeneration, and that deregulation of YAP may play a role in neoplastic transformation and stellate cell functions in PDAC.

YAP protein expression was high in pancreatic cancer cells, and the immunofluorescence results showed that YAP is located in the nucleus and the cytoplasm, but the nuclear expression was stronger than the cytoplasmic expression, which was consistent with a previous study [29]. We found that knockdown of YAP expression via shRNA lentivirus transfection inhibited the proliferation, colony formation and invasion ability of pancreatic cancer cells. EMT is described as a dynamic and reversible biological process, and increasing evidence suggests that EMT plays important roles in the progression of cancer and may provide a rationale for developing more effective cancer therapies [30]. The EMT program is characterized by Vimentin and N-cadherin expression and E-cadherin suppression, representing a highly invasive and mesenchymal phenotype [31]. Consistent with the previous studies [32, 33], our results showed that knockdown of YAP inhibited pancreatic cancer cell invasion, and this was accompanied by a dramatic reduction in the Vimentin and N-cadherin mRNA and protein levels, whereas both E-cadherin mRNA and protein levels were notably increased. It is possible that YAP is a cofactor that interacts with other genes that mediate the EMT process and invasion.

Several studies have suggested that the tumor microenvironment plays a supportive role in PDAC progression [2, 34–36]. During cancer initiation and development, quiescent PSCs are transformed into an activated myofibroblast-like phenotype that is characterized by α-SMA expression and the production of excessive ECM proteins [37]. Increasing evidence suggests that activated PSCs create a stroma-rich and hypoxic microenvironment that facilitates PDAC tumor growth, metastatic spread, perineural invasion, and resistance to chemoradiotherapy [35, 38, 39]. Our IHC results showed that there is a positive correlation between YAP expression and the desmoplastic reaction in human pancreatic cancer tissues and a KPC mouse model. Activation of PSCs was inhibited when they were in a co-culture of PSCs and BxPC-3-shYAP PCs. The α-SMA and TGF-β1 expression in PSCs was down-regulated, and this may be due to the reduction in CTGF, which is a target of YAP and plays important role in PSC activation. In vivo experiments revealed that mice injected with a mixed BxPC-3-shNC+PSC suspension grew larger tumors with extensive desmoplasia and exhibited an increased proliferation tendency compared with mice injected with BxPC-3-shNC cells alone or a mixed BxPC-3-shYAP+PSC suspension. With respect to a mechanism, knockdown of YAP decreases CTGF production and release from PCs, blocking paracrine-mediated PSC activation, and disrupts tumor-stroma interactions.

TGF-β1 is a versatile cytokine that regulates a variety of biological processes through Smad-dependent signaling [40]. TGF-β1 was the first inducer of EMT described in normal mammary epithelial cells, and it was shown to act by signaling through its receptor serine/threonine kinase complex [41]. Currently, it has been documented that cancer cells exhibit increased invasion and metastasis abilities in response to TGF-β1 in various cancer types [42, 43]. Herein, our study showed that TGF-β1 could increase PC invasion and migration by inducing EMT, and this effect was reversed when YAP was knocked down. Western blot showed that knockdown of YAP did not affect the TGF-β1-induced p-SMAD2 level, but the immunofluorescence results showed that the TGF-β1-induced SMAD2 nuclear localization could be reversed when YAP was knocked down. More interestingly, we also found that YAP was significantly up-regulated after treatment with TGF-β1 in a time-dependent and dose-dependent manner, and this up-regulation was reversed after treatment with the TGF-β1 receptor inhibitor SB431542. Taken together, our observations indicate that YAP is a critical mediator of TGF-β1-induced tumorigenic events, including EMT, cell migration, and invasion. Knockdown of YAP disrupted tumor-stroma interactions via reduction of the TGF-β1 production by PSCs and may be the main underlying mechanism of these effects.

## Conclusions

The current study demonstrates that YAP was up-regulated in pancreatic cancers and plays an important role in tumor proliferation, migration and invasion in vitro by modulating EMT-related factors. Knockdown of YAP decreases CTGF production and release from PCs, blocking paracrine-mediated PSC activation and in turn disrupting TGF-β1-mediated tumor-stroma interactions. Thus, YAP may play an important role in EMT and represent a promising therapeutic target for preventing pancreatic cancer progression. In particular, the development of a YAP inhibitor may provide a new class of potent and selective anticancer agents.

## Additional file

Additional file 1: **Table S1.** A list of the utilized primary antibodies. **Table S2.** Primers sequences for real-time PCR analysis. (DOCX 18 kb)

## Abbreviations

CTGF: Connective tissue growth factor
ECM: Extracellular matrix
EMT: Epithelial-mesenchymal transition
FGF: Fibroblast growth factor
KC: LSL-Kras^G12D/+^; Pdx1-Cre
KPC: LSL-Kras^G12D/+^; Trp53^fl/+^; Pdx1-Cre
MMP: Metalloproteinase
PCs: Pancreatic cancer cells
PDAC: Pancreatic ductal adenocarcinoma
PSC: Pancreatic stellate cells
SHH: Sonic Hedgehog
TGF-β1: Transforming growth factor-β1
YAP: YES-associated protein
