# Supplementary material for: Inhibiting YAP expression suppresses pancreatic cancer progression by disrupting tumor-stromal interactions
Source: J Exp Clin Cancer Res. 2018 Mar 27;37:69. doi: 10.1186/s13046-018-0740-4 (PMC5870346; doi:10.1186/s13046-018-0740-4)
Supplement: Supplementary file 1 — Table S1. A list of the utilized primary antibodies. Table S2. Primers sequences for real-time PCR analysis. (DOCX 18 kb) [file 13046_2018_740_MOESM1_ESM.docx]

**Table S1:** A list of the utilized primary antibodies.

| **Antibody** | **Dilution &Use** | **Company** |
| --- | --- | --- |
| Rabbit anti-YAP | 1:2000(WB)  1:150(IF/IHC) | Abcam |
| Rabbit anti-E-cadherin | 1:1000(WB) | Cell Signaling Technology |
| Rabbit anti-N-cadherin | 1:1000(WB) | Cell Signaling Technology |
| Rabbit anti-Vimentin | 1:1000(WB) | Cell Signaling Technology |
| Rabbit anti-SMAD2 | 1:1000(WB)  1:150(IF/IHC) | Proteintech |
| Rabbit anti-p-SMAD2 | 1:1000(WB) | Abcam |
| Mouse anti-α-SMA | 1:1000(WB)  1:150(IF/IHC) | Boster |
| Mouse anti-CTGF | 1:1000(WB)  1:150(IF/IHC) | Boster |
| Mouse anti-PCNA | 1:150(IHC) | Proteintech |
| Mouse anti-TGF-β1 | 1:1000(WB) | RD |
| Mouse anti-β-actin | 1:10000(WB) | Sigma |
| Goat anti-rabbit IgG-HRP | 1:10000(WB) | Abbkine. Inc |
| Goat anti-mouse IgG-HRP | 1:10000(WB) | Abbkine. Inc |
| Goat anti-rabbit dylight 594  (red) IgG antibody | 1:150 (IF) | Abbkine. Inc |
| Goat anti-mouse dylight 488  (green) IgG antibody | 1:150 (IF) | Abbkine. Inc |

**Table S2:** Primers sequences for real-time PCR analysis.

| **Genes** | **Primer Sequences** |
| --- | --- |
| YAP | Forward:5’-TCCCAGATGAACGTCACAGC-3’ |
|  | Reverse: 5’-TCATGGCAAAACGAGGGTCA-3’ |
| E-cadherin | Forward:5’-ATTCTGATTCTGCTGCTCTTG-3’ |
|  | Reverse: 5’-AGTCCTGGTCCTCTTCTCC-3’ |
| N-cadherin | Forward:5’-ACAACAGACCTGAGTTCTTACAC-3’ |
|  | Reverse: 5’-TTGGAGCCTGAGACACGATT-3’ |
| Vimentin | Forward:5’-AATGACCGCTTCGCCAAC-3’ |
|  | Reverse: 5’-CCGCATCTCCTCCTCGTAG-3’ |
| CTGF | Forward:5’-CTTTGGCCCAGACCCAACTA-3’ |
|  | Reverse: 5’-GGCTCTGCTTCTCTAGCCTG-3’ |
| β-actin | Forward:5’- AGCGAGTATCCCCCAAAGTT-3’ |
|  | Reverse: 5’- GGGCACGAAGGCTCATCATT-3’ |
